# Structural error asymmetry and harm-weighted analysis of ChatGPT versus ICU Physicians in acid–base interpretation: a prospective observational study

**DOI:** 10.1038/s41598-026-44576-4

**Published:** 2026-03-27

**Authors:** Derful Gulen, Hilmi Erdem Gözden, Serpil Ekin, Ilkay Ceylan

**Affiliations:** 1 Department of Anesthesiology and Reanimation, University of Health Sciences, Bursa Yüksek İhtisas Training and Research Hospital, Bursa, Türkiye Turkey; 2 Department of Hematology, University of Health Sciences, Bursa Yüksek İhtisas Training and Research Hospital, Bursa, Türkiye Turkey

**Keywords:** Computational biology and bioinformatics, Diseases, Health care, Medical research

## Abstract

Large language models (LLMs) have demonstrated potential in clinical reasoning tasks; however, their performance in real-world intensive care unit (ICU) acid–base interpretation remains insufficiently characterized, particularly in complex and mixed disorders. Most existing evaluations rely primarily on aggregate accuracy metrics without examining structural error patterns or the clinical severity of misclassification. In high-acuity ICU settings, under-recognition of physiological complexity may carry disproportionate safety implications. In this prospective observational study, arterial blood gas (ABG) data from 50 consecutive ICU patients were interpreted independently by ICU physicians and ChatGPT using a standardized prompt. Interpretations were harmonized into six predefined diagnostic categories and compared with a final reference diagnosis established by a blinded expert panel. Agreement was assessed using Cohen’s kappa and diagnostic accuracy metrics. Additional analyses included complexity-stratified evaluation, mixed-disorder sensitivity, multi-label component-level (metabolic and respiratory) detection, false reassurance risk assessment, harm-weighted misclassification modeling, bootstrap confidence intervals, and post-hoc power analysis. Overall categorical accuracy was 82% for ICU physicians and 72% for ChatGPT. Agreement was substantial in both groups (κ = 0.73 vs. 0.63). Paired comparison did not demonstrate a statistically significant difference in overall classification (*p* = 0.267), and post-hoc power analysis indicated limited ability to detect modest effect sizes (power = 0.22). However, stratified analyses revealed clinically meaningful structural differences. Sensitivity for mixed acid–base disorders was 0.96 for ICU physicians and 0.63 for ChatGPT. ChatGPT uniquely classified 16.7% of mixed cases as normal (false reassurance), whereas ICU physicians produced no false-normal classifications. Component-level analysis demonstrated lower respiratory component sensitivity for ChatGPT (0.88 vs. 1.00), contributing to under-recognition of physiological complexity. Harm-weighted misclassification modeling showed significantly higher clinical severity of errors for ChatGPT (mean difference 0.12; 95% CI 0.032–0.220; *p* = 0.026). While aggregate diagnostic agreement appeared broadly comparable, complexity-stratified and harm-weighted analyses demonstrated asymmetric error patterns with potential safety implications. These findings do not establish clinical equivalence and suggest that evaluation of AI diagnostic tools in critical care should extend beyond overall accuracy to incorporate safety-oriented and harm-weighted assessment frameworks.

**Supplementary Information:**

The online version contains supplementary material available at 10.1038/s41598-026-44576-4.

## Introduction

Arterial blood gas (ABG) analysis is a critical skill in intensive care units (ICUs) and emergency settings, providing insight into oxygenation, acid-base balance, and metabolic status^1^. However, ABG interpretation can be challenging and subject to human error, especially in time-pressured critical care scenarios^2^. Variability in clinician expertise may lead to misinterpretation or delayed recognition of complex acid-base disturbances^3^.

Artificial intelligence (AI) has rapidly entered clinical medicine, with large language models (LLMs) like ChatGPT showing potential to assist in diagnosis and decision-making^4^. The advent of advanced LLMs has raised the question of whether AI can augment or rival human performance in structured interpretative tasks^5^. Since its public launch in late 2022, ChatGPT has been explored for diverse medical applications including clinical diagnostics, patient management support, and medical education^6^.

Several studies have examined ChatGPT’s abilities in critical care medicine, where timely interpretation of structured physiological data such as ABGs is essential^7^. ABG interpretation represents a compelling test case for LLM evaluation because it involves standardized input parameters (pH, PaCO₂, HCO₃⁻) and established diagnostic frameworks for acid–base disorders. Studies have demonstrated that ChatGPT can achieve high accuracy in classifying basic parameters such as pH and oxygenation status^8^.

However, performance consistency appears to decline with increasing physiological complexity. ChatGPT demonstrates strong performance on straightforward cases but reduced reliability in mixed acid–base disorders typical of critically ill ICU populations^9^. Importantly, most prior comparisons have relied primarily on aggregate accuracy or agreement metrics, without examining structural error patterns, diagnostic decomposition, or the clinical severity of misclassification.

In high-acuity ICU environments, the consequences of under-recognizing physiological complexity—particularly mixed disorders—may exceed those of simple categorical disagreement. Therefore, evaluation frameworks that incorporate component-level detection and harm-weighted misclassification analysis may provide a more clinically meaningful assessment than overall agreement statistics alone.

This study was designed not only to compare overall diagnostic agreement between ChatGPT and ICU physicians, but also to characterize differences in error topology, complexity recognition, and harm-weighted misclassification patterns.

This study aimed to compare the diagnostic performance of ChatGPT and intensive care unit physicians in the interpretation of case-based acid–base disorders, using structured clinical vignettes derived from real ICU patients and the final ICU diagnosis as the reference standard within a single-center prospective observational design.

## Methods

Rather than isolated arterial blood gas values, both ChatGPT and clinicians were provided with structured case-based clinical scenarios incorporating arterial blood gas results and relevant contextual clinical information.

### Study design and setting

This study was designed as a single-center, prospective observational comparison of arterial blood gas (ABG) interpretation performance between intensive care unit (ICU) physicians and a large language model (ChatGPT). The study was conducted in the adult ICU of Bursa Yüksek İhtisas Training and Research Hospital, a tertiary referral center. All cases were derived from real ICU patients and prospectively collected during routine clinical care; no simulated or hypothetical cases were used.

The study protocol was approved by the University of Health Sciences Bursa Yüksek İhtisas Training and Research Hospital Ethics Committee of Medical Sciences (approval number: 2024-TBEK-2025/07–11, July 16, 2025). All procedures were conducted in accordance with the Declaration of Helsinki.

### Study population and patient inclusion criteria

Patients were prospectively enrolled between August 2025 and September 2025 in the adult intensive care unit of Bursa Yüksek İhtisas Training and Research Hospital.

During this period, 50 consecutive adult ICU patients who met the predefined eligibility criteria were included in the study.

Inclusion criteria were:

• age ≥ 18 years,
• availability of a complete arterial blood gas analysis,
• ICU admission requiring clinical ABG interpretation.

Exclusion criteria were:

• incomplete ABG data,
• technically invalid samples,
• repeat ABG measurements from the same patient.

Only the first qualifying ABG sample per patient was included to avoid intra-individual correlation.

The sample size was determined by the number of consecutive eligible ICU admissions during the predefined study period rather than by a priori power calculation.

Given the exploratory and feasibility-oriented nature of the study, no formal sample size calculation was performed before enrollment. A post-hoc power analysis based on observed effect sizes was conducted to contextualize inferential findings.

### Data collection and ABG variables

For each patient, arterial blood gas parameters (pH, PaCO₂, PaO₂, HCO₃⁻, base excess, oxygen saturation, FiO₂) were extracted from the electronic medical record.

Additional clinical and laboratory variables relevant to acid–base interpretation (including lactate, serum electrolytes, renal function parameters, ventilatory settings, and hemodynamic support) were collected using a standardized data extraction form. Severity-of-illness scores (APACHE II and SOFA) were recorded for descriptive and exploratory analyses but were not provided to ChatGPT.

### ChatGPT access and model version

Artificial intelligence assistance was obtained via the publicly available ChatGPT web interface (OpenAI, San Francisco, CA, USA) using a ChatGPT Plus subscription. During the study period (August–September 2025), GPT-4o, a GPT-4–class large language model, was utilized. Model access occurred through the standard web interface without API-based customization. Exact internal version identifiers were not accessible to the investigators. No parameter tuning, system-level configuration, or temperature modification was performed.

### Diagnostic category harmonization

To enable standardized comparison across evaluators, all diagnostic interpretations were harmonized into six predefined categories: normal acid–base status, metabolic acidosis, metabolic alkalosis, respiratory acidosis, respiratory alkalosis, and mixed acid–base disorder.

Free-text interpretations from ICU physicians and ChatGPT were independently mapped to these categories according to predefined operational definitions. This harmonization step was performed prior to statistical analysis and blinded to reference standard outcomes. The ICU physician group consisted of three clinicians with heterogeneous training backgrounds (internal medicine and anesthesiology) and varying levels of experience (residents, fellows, and attending physicians).

The median ICU experience among clinicians was 12 years. Individual experience levels were 6, 12, and 20 years, reflecting a heterogeneous group of physicians across different training levels.

### Complexity stratification

Cases were stratified according to final reference diagnosis into single (primary) acid-base disorders and mixed acid–base disorders. The terms “simple” and “isolated” were not used in the final analysis to avoid terminological ambiguity. Complexity-stratified analyses were pre-specified to evaluate differential performance in physiologically complex cases.

### Multi-Label component-level analysis

Because acid–base disorders represent combinations of metabolic and respiratory physiological processes, a multi-label component analysis was conducted in addition to categorical classification.

For each case, presence or absence of a metabolic component and a respiratory component was coded independently. Sensitivity and specificity for detection of metabolic and respiratory contributions were calculated separately using the final reference diagnosis as ground truth.

This approach was implemented to mitigate limitations associated with discrete categorical classification of continuous physiological processes.

### False reassurance definition

False reassurance was predefined as classification of a mixed acid–base disorder as normal acid–base status. This misclassification was considered clinically high-severity due to potential implications for delayed escalation in critical care settings.

### Harm-Weighted misclassification modeling

To quantify clinical severity of diagnostic errors beyond categorical accuracy, a harm-weighted misclassification model was applied at the case level.

Predefined harm weights were assigned to each misclassification type based on a severity hierarchy, with highest weight assigned to false reassurance in mixed disorders, followed by misclassification of mixed as single (primary) disorders, and lowest weight assigned to intra-class substitutions.

For each evaluator, a mean harm score was calculated across all cases. Paired comparison between ICU physicians and ChatGPT was performed using the Wilcoxon signed-rank test.

Bootstrap resampling (5,000 iterations) with percentile-based confidence intervals was used to estimate the 95% confidence interval of the mean harm difference.

### Clinical input structure provided to ChatGPT

For each patient, ChatGPT received a standardized, structured clinical vignette prepared in English using prospectively collected ICU data. The full set of clinical, laboratory, and arterial blood gas variables included in the vignette is detailed in Table 1.

**Table 1:** Standardized Clinical Input Structure and Variable Domains Provided to ChatGPT.

Domain	Variables Included	Purpose in ABG Interpretation
Model Instruction (Prompt)	*“You are an intensive care specialist… including identification of the primary acid–base disorder and its likely cause.”*	Standardize expert-level clinical reasoning and eliminate prompt variability
Demographics	Age, Sex	Contextual risk stratification and physiologic baseline
ICU Admission Context	Reason for ICU admission	Identification of underlying pathophysiology affecting acid–base balance
Respiratory Support	Mechanical ventilation status; mode; FiO₂; PEEP; respiratory rate; tidal volume	Interpretation of respiratory compensation and ventilatory contribution
Hemodynamic Support	Vasopressor use; sedation status	Assessment of shock-related metabolic disturbances
Physical Examination	GCS; vital signs; lung auscultation; capillary refill time	Integration of clinical severity and perfusion status
Comorbid Conditions	Chronic kidney disease; diabetes mellitus; hypertension; other relevant comorbidities	Identification of predisposition to metabolic derangements
Laboratory Parameters	Hemoglobin; WBC; platelets; Na⁺; K⁺; Cl⁻; serum HCO₃⁻; urea; creatinine; glucose; AST; ALT; bilirubin; albumin; CRP; lactate	Identification of metabolic, renal, and inflammatory contributors
Arterial Blood Gas Variables	pH; PaCO₂; PaO₂; HCO₃⁻; base excess; oxygen saturation; FiO₂	Core variables for acid–base disorder classification
Output Requested	Primary disorder; compensation/mixed disorder; likely etiology	Structured diagnostic interpretation for categorical comparison

All clinical inputs were provided to ChatGPT in English using a fixed prompt and standardized structure. The prompt and variable set were identical for all cases and were not modified during the study period.

### Diagnostic assessment workflow

Each ABG case underwent three independent interpretations:

1. Initial interpretation by the attending ICU physician,
2. Interpretation by ChatGPT using a standardized prompt and identical structured input,
3. Final reference diagnosis established by a blinded expert panel.

All interpretations were performed independently, and evaluators were blinded to each other’s assessments. Detailed information regarding the composition, clinical roles, and professional experience of the expert panel is provided in Supplementary Appendix B.

### Statistical analysis

All statistical analyses were performed using R Studio (R version 4.4.3). Descriptive statistics were calculated for demographic and clinical variables. Diagnostic accuracy was calculated as the proportion of cases in which the interpretation matched the predefined reference standard. Agreement was assessed using Cohen’s kappa (κ) with 95% confidence intervals. Paired discordance between ICU physicians and ChatGPT was evaluated using McNemar’s test.

Component-level sensitivity and specificity were calculated independently for metabolic and respiratory detection using standard binomial estimators. Exact binomial confidence intervals were computed for false reassurance rates in mixed disorders. Harm-weighted differences were evaluated using paired non-parametric testing and bootstrap confidence interval estimation.

A post-hoc power analysis based on observed effect size (Cohen’s h) was performed using two-proportion comparison to estimate achieved statistical power. All statistical tests were two-sided. A p-value < 0.05 was considered statistically significant. No adjustment for multiple comparisons was applied, as secondary analyses were exploratory and hypothesis-generating.

## Results

Descriptive characteristics of the study population and overall diagnostic performance are presented in Tables. Figures are reserved for key comparative and structural error analyses.

### Study population characteristics

The study included 50 adult ICU patients. The age distribution was right-skewed, with a median age of 71.5 years (IQR 55.8–80). The Shapiro–Wilk test indicated non-normal distribution (*p* = 0.023). The cohort consisted of 28 male patients (56%) and 22 female patients (44%). APACHE II scores ranged from 5 to 50 (median 24), and SOFA scores ranged from 2 to 12 (median 6). A moderate positive correlation was observed between APACHE II and SOFA scores (Spearman ρ = 0.47, *p* < 0.001), reflecting internal consistency of severity assessment tools (Table 2).

**Table 2:** Baseline characteristics of the study population (*n* = 50).

Characteristic	Value
Age, years, median (IQR)	71.5 (55.8–80.0)
Sex, n (%)	
Male	28 (56%)
Female	22 (44%)
APACHE II score, median (IQR)	24.0 (19.0–25.8)
SOFA score, median (IQR)	6.0 (4.0–8.0)
Mechanical ventilation, n (%)	
Yes	33 (66%)
No	17 (34%)
Primary reason for ICU admission, n (%)	
Respiratory causes (e.g., pneumonia, respiratory failure)	≈ 28%*
Sepsis / infection-related	≈ 24%*
Cardiovascular conditions	≈ 20%*
Neurological conditions	≈ 16%*
Other causes	≈ 12%*

Data are presented as median (interquartile range) or number (percentage), as appropriate.

APACHE II: Acute Physiology and Chronic Health Evaluation II;

SOFA: Sequential Organ Failure Assessment;

ICU: intensive care unit.

*Percentages for ICU admission categories are rounded and based on grouped clinical diagnoses.

The primary reasons for ICU admission are summarized in Table 3. The most frequent indications were respiratory failure (28%) and sepsis/septic shock (24%), followed by cardiovascular conditions (20%) and neurological disorders (16%). Other causes accounted for 12% of admissions. This distribution reflects a high-acuity ICU population with a predominance of conditions commonly associated with complex acid–base disturbances (Table 3).

**Table 3:** Primary reasons for ICU admission.

Admission category	*n* (%)
Respiratory causes	14 (28%)
Sepsis / infection-related	12 (24%)
Cardiovascular conditions	10 (20%)
Neurological conditions	8 (16%)
Other causes	6 (12%)

### Distribution of final acid–base diagnoses

After category harmonization into six standardized diagnostic groups, mixed acid–base disorders represented 48% of cases (24/50), metabolic alkalosis 16% (8/50), respiratory alkalosis 12% (6/50), normal status 10% (5/50), respiratory acidosis 8% (4/50), and metabolic acidosis 6% (3/50).

The high proportion of mixed disorders reflects the physiological complexity typical of critically ill ICU populations and provides a clinically relevant test environment for diagnostic decomposition analysis.

### Overall diagnostic accuracy and agreement

Overall diagnostic accuracy was 82% (41/50) for ICU physicians and 72% (36/50) for ChatGPT (Table 4).

**Table 4:** Diagnostic accuracy of ICU physicians and ChatGPT.

Evaluator	Correct diagnoses, *n* (%)	Incorrect diagnoses, *n* (%)
ICU physician	41 (82%)	9 (18%)
ChatGPT	36 (72%)	14 (28%)

Paired comparison using McNemar’s test showed no statistically significant difference (*p* = 0.267).

Bootstrap-derived 95% confidence intervals for overall accuracy overlapped between groups.

Agreement with the final reference diagnosis was substantial for both evaluators:

- ICU physicians: κ = 0.73.
- ChatGPT: κ = 0.63.

Paired comparison using the McNemar test showed no statistically significant difference in overall correct versus incorrect classifications (*p* = 0.267).

Post-hoc power analysis based on observed effect size (Cohen’s h = 0.24) indicated achieved power of 0.22, underscoring the exploratory nature of inferential comparisons.

### Complexity-stratified performance

**Fig. 1:**
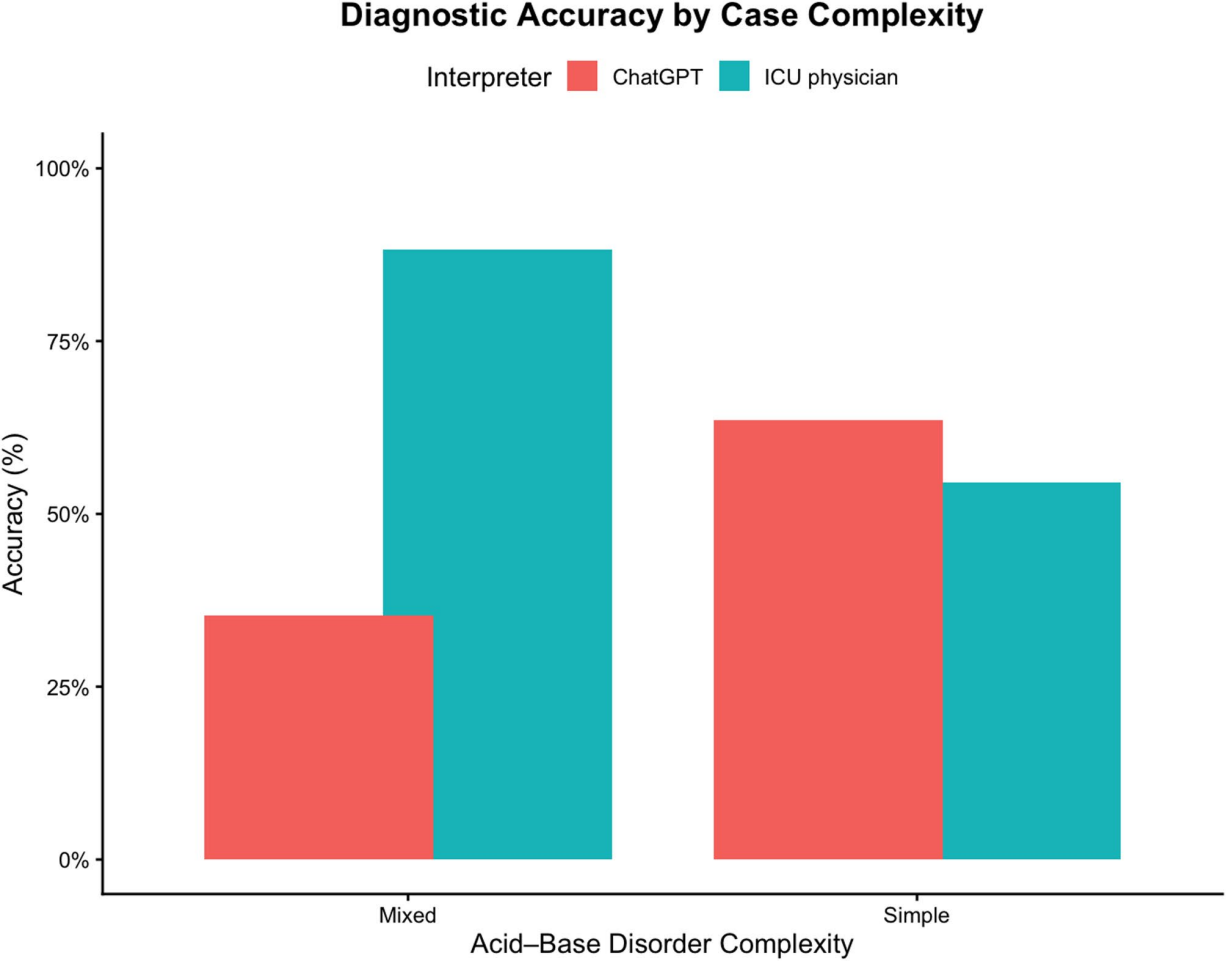
Subgroup analysis of diagnostic performance by case complexity (single [primary] disorders vs. mixed acid–base disorders)

Performance differed substantially according to case complexity (Figure 1). Sensitivity for mixed acid–base disorder detection was 0.96 (23/24) for ICU physicians and 0.63 (15/24) for ChatGPT (Table 5). Specificity for mixed disorders was 0.73 for ICU physicians and 0.88 for ChatGPT.

**Table 5:** Component-Level Detection.

Metric	ICU (*n*/*N*)	ChatGPT (*n*/*N*)
Mixed sensitivity	23/24 (0.96)	15/24 (0.63)
Mixed specificity	19/26 (0.73)	23/26 (0.88)
Metabolic sensitivity	34/35 (0.97)	31/35 (0.89)
Respiratory sensitivity	34/34 (1.00)	30/34 (0.88)
False-normal in mixed	0/24 (0%)	4/24 (16.7%)

Diagnostic performance metrics for detection of metabolic and respiratory components of acid–base disorders using multi-label component analysis. Sensitivity and specificity were calculated independently for metabolic and respiratory contributions using the final ICU diagnosis as reference standard. False-negative counts are provided to illustrate component under-recognition patterns. This analysis was performed to address limitations inherent in discrete categorical classification of continuous physiological processes and to evaluate structural differences in diagnostic decomposition between ICU physicians and ChatGPT.

**Fig. 2:**
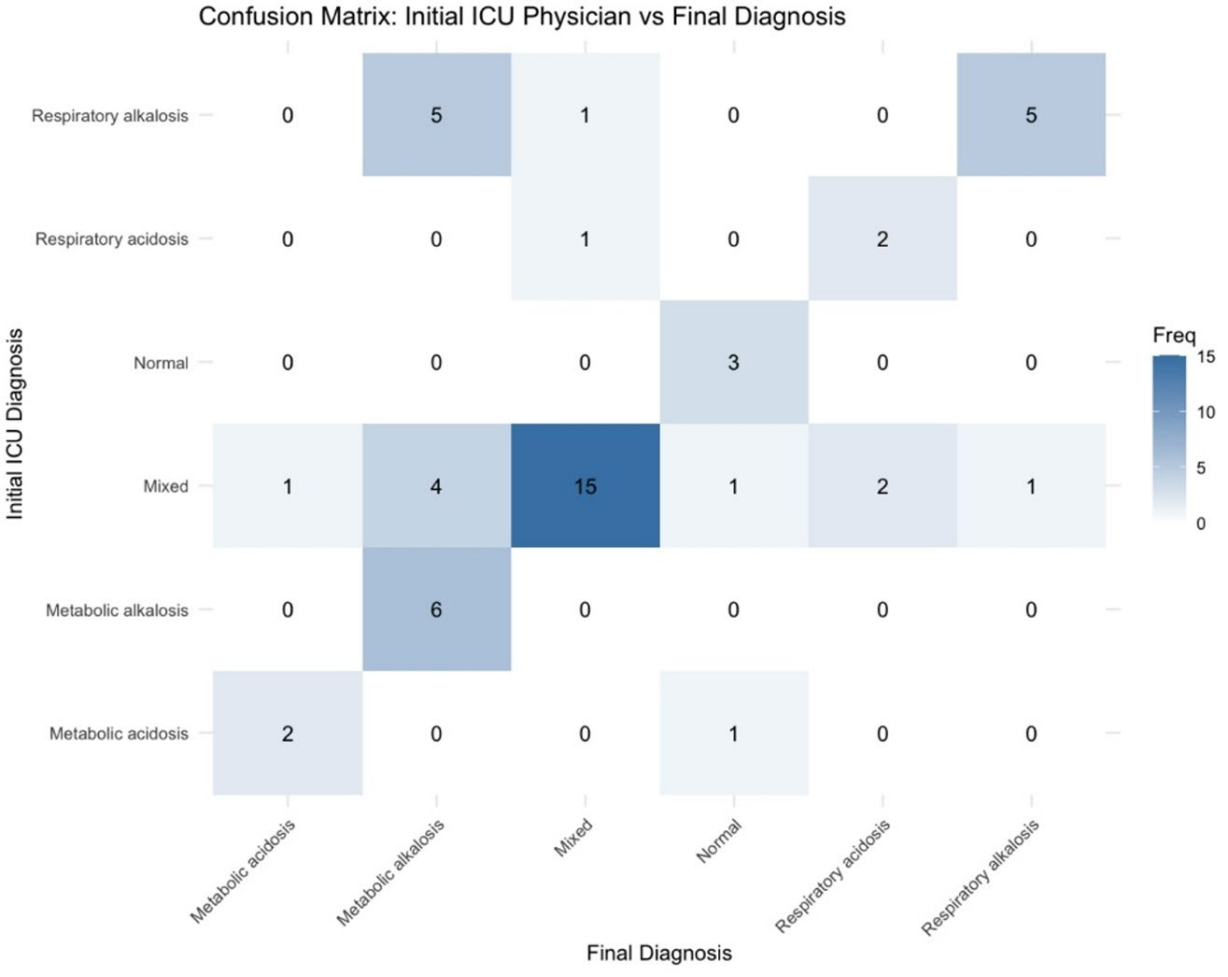
Mixed Acid-Base Disorder Detection *(Error structure comparison between ICU physicians and ChatGPT).* Stacked bar plot depicting classification outcomes among cases with a final diagnosis of mixed acid–base disorder (*n* = 24). Categories include correct identification (“Correct”), misclassification as a single (primary) disorder (“Missed-Single (primary)”), and misclassification as normal acid–base status (“False Normal”). ICU physicians demonstrated high sensitivity for mixed disorder detection and produced no false-normal classifications. ChatGPT showed reduced mixed-detection sensitivity and uniquely classified a subset of mixed cases as normal, indicating a false reassurance pattern. Bar proportions are calculated relative to the total number of cases with final mixed acid–base diagnosis (*n* = 24).

ChatGPT uniquely classified 16.7% (4/24) of mixed disorders as normal acid–base status (false reassurance), whereas ICU physicians produced no false-normal classifications (Fig. 2).

Exact binomial analysis confirmed that the false-normal rate among mixed cases was significantly different from zero for ChatGPT (*p* = 0.0015).

### Component-level detection analysis

To examine structural differences in diagnostic decomposition, metabolic and respiratory components were analyzed independently.

Metabolic component sensitivity:

- ICU physicians: 0.97 (34/35).
- ChatGPT: 0.89 (31/35).

Respiratory component sensitivity:

- ICU physicians: 1.00 (34/34).
- ChatGPT: 0.88 (30/34).

**Fig. 3:**
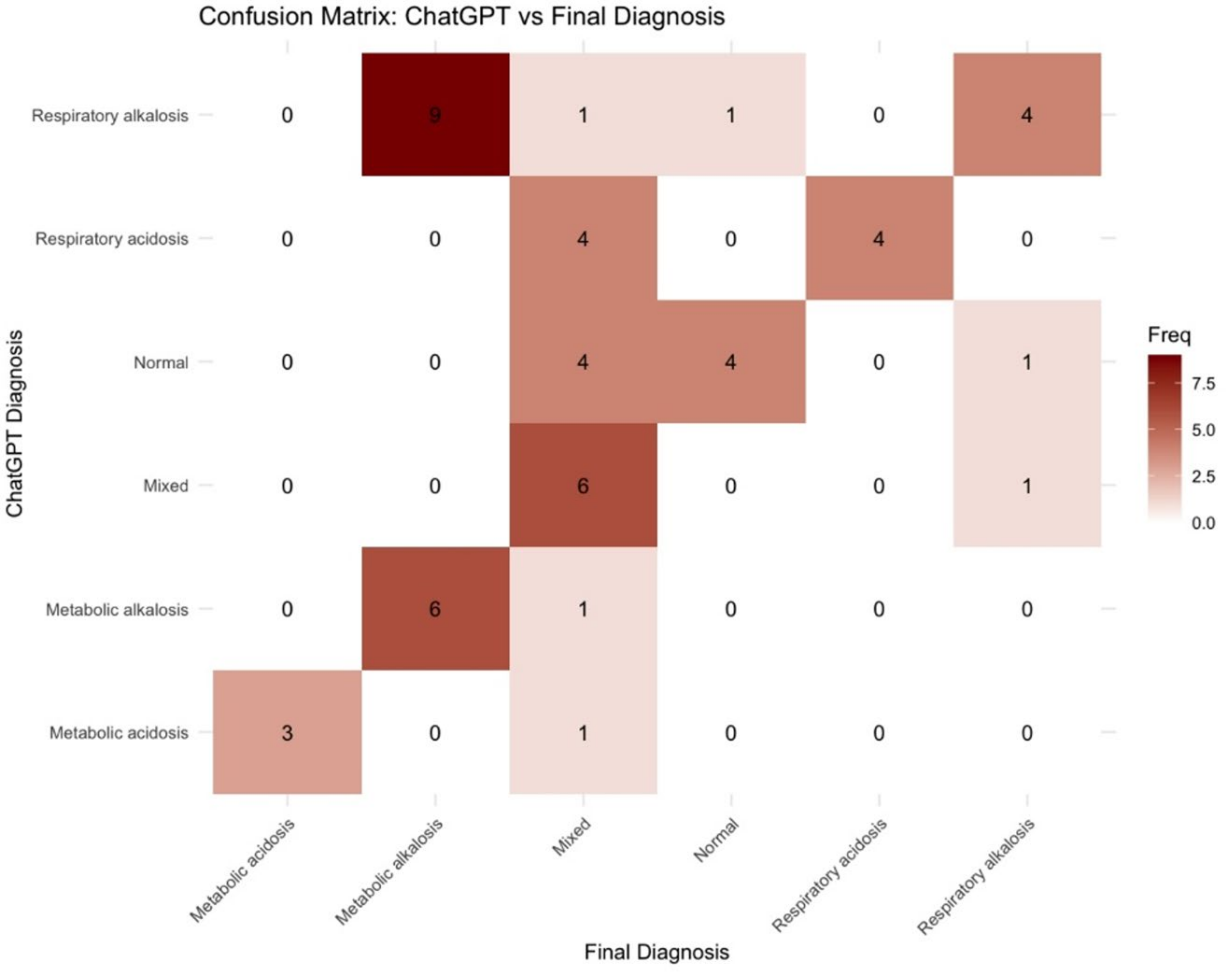
Component Level Detection Sensitivity *(Comparison of metabolic and respiratory component identification).* Bar graph comparing sensitivity for identification of metabolic and respiratory components of acid–base disorders between ICU physicians and ChatGPT. Sensitivity was calculated independently for metabolic and respiratory contributions using multi-label component analysis. While metabolic component detection showed relatively small differences between groups, respiratory component sensitivity was lower for ChatGPT, contributing to under-recognition of mixed acid–base disorders. Sensitivity values represent true positive rate relative to the total number of cases with the corresponding metabolic or respiratory component in the reference diagnosis.

Reduced respiratory component sensitivity in ChatGPT contributed to under-recognition of mixed acid–base disorders (Table 5) (Fig. 3).

**Fig. 4:**
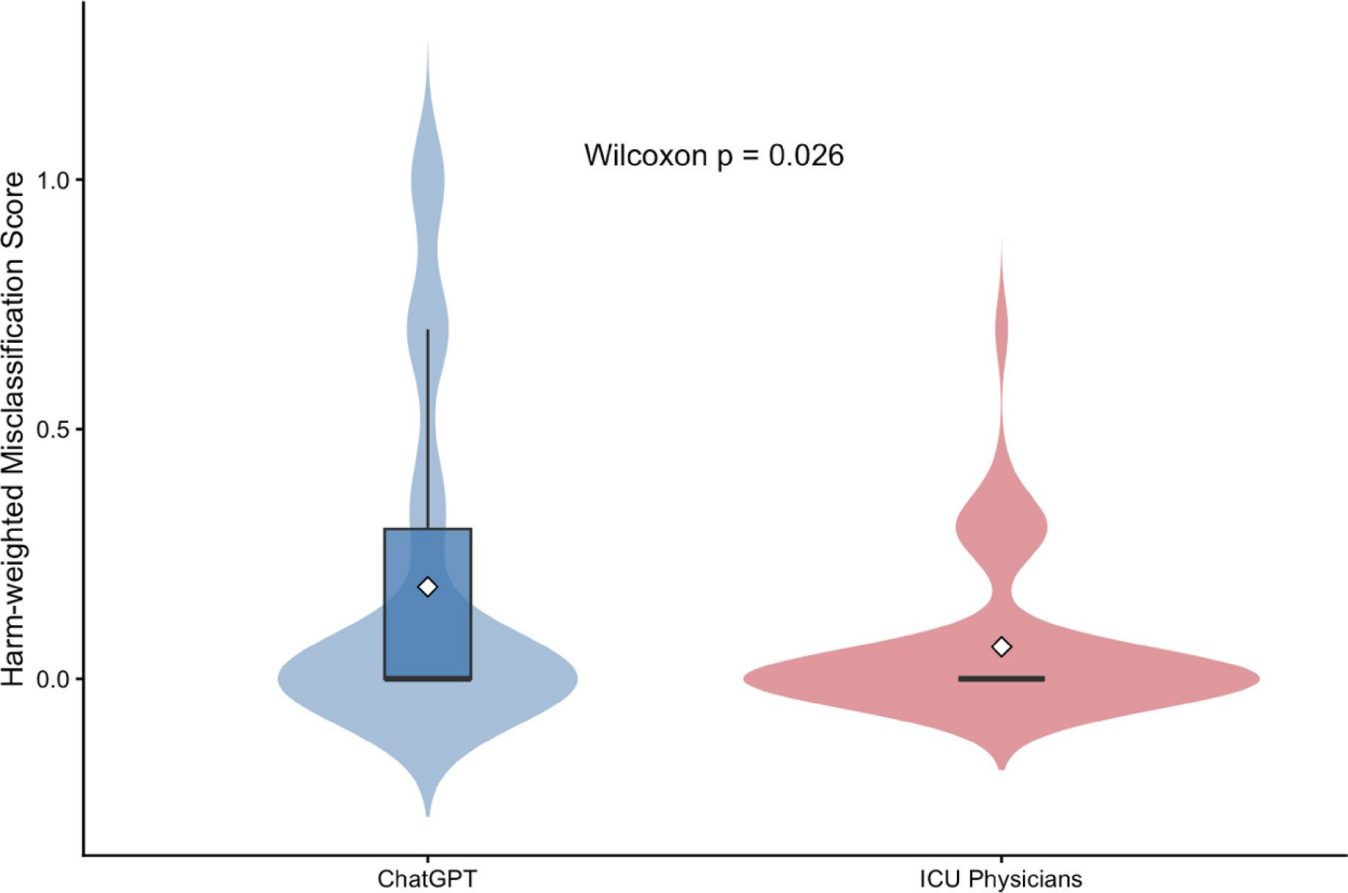
Distribution of Harm-Weighted Diagnostic Errors *(Paired comparison between ICU physicians and ChatGPT).* Violin and box plot illustrating the distribution of harm-weighted misclassification scores for ICU physicians and ChatGPT across all cases (*n* = 50). The harm-weighted score assigns increasing values to misclassifications based on estimated clinical severity, with highest weighting applied to false reassurance in mixed acid–base disorders. White diamonds represent mean values. Paired Wilcoxon signed-rank testing demonstrated significantly higher harm-weighted error for ChatGPT compared with ICU physicians (*p* = 0.026). Bootstrap analysis (5,000 resamples) yielded a 95% confidence interval for the mean difference of 0.032–0.220.

### Harm-weighted misclassification analysis

To evaluate clinical severity of diagnostic errors, harm-weighted misclassification scores were calculated at the case level.

Mean harm score:

- ICU physicians: 0.064.
- ChatGPT: 0.184.

Mean paired difference: 0.12.

Paired Wilcoxon signed-rank test demonstrated significantly higher harm scores for ChatGPT (*p* = 0.026). Bootstrap resampling (5,000 iterations) yielded a 95% confidence interval for the mean harm difference of 0.032–0.220, excluding zero (Table 6; Fig. 4).

**Table 6:** Harm-Weighted Analysis.

Metric	ICU	ChatGPT	Mean Difference	95% CI	*p*-value
Mean harm score	0.064	0.184	0.12	0.032–0.220	0.026
Median harm score	0	0	—	—	—

Comparison of harm-weighted misclassification scores between ICU physicians and ChatGPT. Harm scores were assigned at the case level based on predefined clinical severity weighting of misclassification types, with highest weight assigned to false reassurance in mixed acid–base disorders. Mean harm scores, mean paired difference, bootstrap 95% confidence intervals (5,000 resamples), and paired Wilcoxon signed-rank p-values are reported. This analysis was performed to quantify differential clinical risk profiles beyond aggregate categorical accuracy.

### Error topology patterns

Confusion matrix analysis demonstrated divergent structural error patterns.

**Fig. 5:**
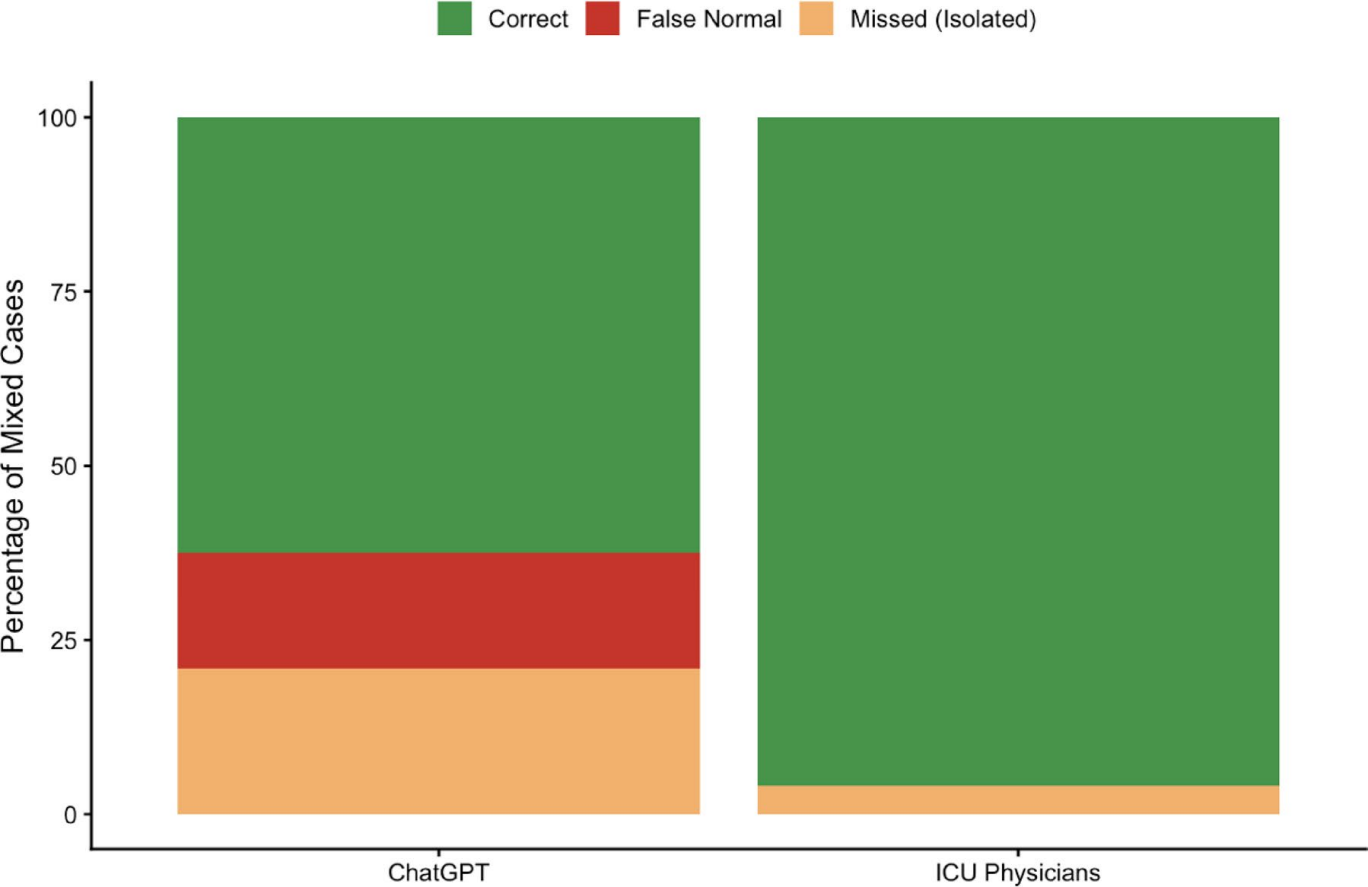
Confusion matrix comparing initial ICU physician diagnoses with final ICU diagnoses. Heatmap representation of diagnostic agreement between the initial ICU physician assessment (rows) and the final ICU diagnosis (columns), which served as the reference standard. Diagonal cells represent correct classifications, whereas off-diagonal cells indicate misclassifications. The matrix demonstrates a high sensitivity for mixed acid–base disorders by ICU physicians, accompanied by a tendency toward overclassification, while metabolic and respiratory disturbances show moderate concordance with the final diagnosis. Percentages displayed in the heatmap represent row-wise proportions (i.e., proportion of each predicted category relative to the total number of cases in that row).

**Fig. 6:**
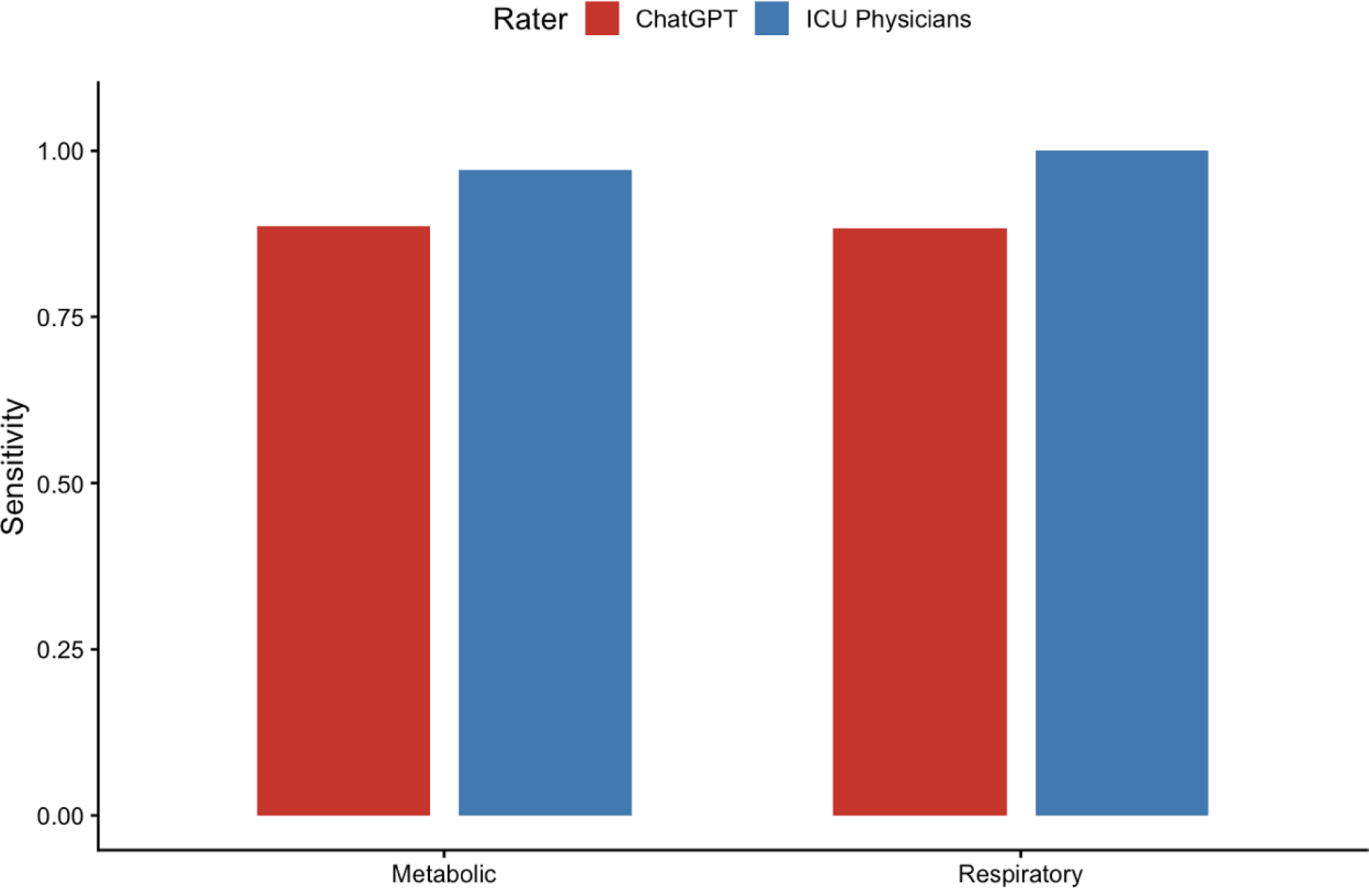
Confusion matrix comparing ChatGPT diagnoses with final ICU diagnoses. Heatmap illustrating agreement between ChatGPT-assigned acid–base diagnoses (rows) and the final ICU diagnosis (columns), which served as the reference standard. Diagonal cells represent correct classifications, whereas off-diagonal cells indicate misclassifications. ChatGPT demonstrates a tendency to classify mixed acid–base disorders as metabolic or respiratory disturbances and to assign normal acid–base status in borderline cases, highlighting differences in diagnostic reasoning compared with human clinicians. Percentages displayed in the heatmap represent row-wise proportions (i.e., proportion of each predicted category relative to the total number of cases in that row).

ICU physicians showed a tendency toward cautious overclassification of complexity (Figure 5), whereas ChatGPT more frequently decomposed mixed disorders into single (primary) categories or normal classifications (Figure 6).

These findings indicate asymmetric error topology between evaluators despite broadly comparable aggregate accuracy metrics.

## Discussion

### Performance contextualization within current literature

In this prospective ICU cohort, overall diagnostic accuracy was 82% (41/50) for ICU physicians and 72% (36/50) for ChatGPT. Although agreement was substantial in both groups (κ = 0.73 vs. 0.63), aggregate metrics alone did not fully capture structural differences in diagnostic behavior.

Previous systematic reviews have reported generative AI diagnostic performance in the range of approximately 50–60% across heterogeneous clinical settings^5,10^. However, many prior studies relied primarily on global accuracy or agreement statistics without stratifying by physiological complexity or quantifying the clinical severity of misclassification.

Our findings extend this literature by demonstrating that comparable aggregate accuracy may coexist with asymmetric error topology, particularly in high-complexity ICU cases.

### Case complexity as a critical determinant

Mixed acid–base disorders represented 48% of cases in our cohort (24/50), reflecting the physiological complexity of critically ill patients. Sensitivity for mixed disorder detection was 0.96 for ICU physicians compared with 0.63 for ChatGPT. This confirms prior observations that performance declines with increasing complexity^3,9^.

Importantly, the discrepancy was not limited to reduced sensitivity but included a distinct false reassurance pattern: ChatGPT classified 16.7% of mixed disorders as normal, whereas ICU physicians produced no false-normal classifications.

This finding refines earlier reports of “complexity under-recognition” by quantifying its clinical expression in real ICU cases.

### Structural diagnostic decomposition

To address limitations of categorical evaluation, metabolic and respiratory components were analyzed independently. Respiratory component sensitivity was 1.00 for ICU physicians and 0.88 for ChatGPT, suggesting incomplete physiological decomposition contributed to mixed-disorder under-recognition.

This structural decomposition analysis goes beyond previous reports attributing errors solely to arithmetic miscalculation^2^. Rather than isolated formula errors, our data suggest a pattern of partial physiological reconstruction under complexity conditions.

### Harm-weighted misclassification and clinical risk

A key extension of the present study is the incorporation of harm-weighted misclassification modeling. Despite broadly comparable overall agreement, mean harm scores were significantly higher for ChatGPT (0.184 vs. 0.064; mean paired difference 0.12; 95% CI 0.032–0.220; *p* = 0.026). This indicates that differences between evaluators were not merely quantitative but qualitatively related to clinical severity of error.

Comparable accuracy should therefore not be interpreted as equivalence in clinical safety, particularly in high-acuity settings where under-recognition of evolving complexity may delay escalation.

### Interpretation of non-significant overall difference

Paired comparison of overall correct classifications was not statistically significant. However, post-hoc power analysis demonstrated limited statistical power (0.22) to detect moderate differences, indicating that absence of statistical significance should not be interpreted as evidence of equivalence. This aligns with broader meta-analytic observations that AI performance frequently approaches but does not surpass specialist-level interpretation^5,10-12^.

### Clinical integration and human oversight

The data do not support autonomous deployment of general-purpose LLMs for independent acid–base interpretation in critical care. At the same time, the results do not suggest universal inferiority; rather, they indicate differential strengths depending on case complexity.

Human-in-the-loop models, in which AI functions as structured analytical support under physician supervision, may represent a more appropriate translational pathway^13^. Importantly, inability to prospectively identify case complexity at the bedside limits the safety of conditional reliance on AI outputs.

### Temporal dynamics and real-world decision-making

This study evaluated single time-point case-based assessments and did not incorporate longitudinal physiological trends, treatment response, or escalation behavior^7^.

Because ICU decision-making is trajectory-based rather than static, future evaluations should incorporate temporal modeling to better approximate real-world clinical reasoning.

### Limitations

This study has several limitations. First, the sample size (*n* = 50) limits precision of subgroup estimates. Second, ChatGPT outputs did not provide calibrated uncertainty or probabilistic confidence estimates, precluding formal calibration analysis. Third, single-center design may limit generalizability. The study utilized a GPT-4–based model accessible between August and September 2025; however, granular internal version identifiers and backend update logs were not available to investigators. Given the dynamic update structure of large language models, performance may vary across future iterations^14^. Fourth, the final reference diagnosis was established by an expert panel using case-based vignette review; although panelists were not involved in bedside care of the included patients and did not have access to longitudinal clinical trajectories or outcomes, reference standard adjudication may still introduce subjectivity compared with a fully protocolized algorithmic standard.

### Implications for evaluation frameworks

Our findings suggest that evaluation of AI diagnostic tools in critical care should extend beyond aggregate accuracy to include:

- complexity-stratified analysis,
- component-level physiological decomposition,
- false reassurance quantification,
- and harm-weighted error modeling.

Such safety-oriented frameworks may better characterize clinical risk profiles than agreement statistics alone.

## Conclusion

In this prospective, real-world ICU cohort, ChatGPT demonstrated broadly comparable overall categorical diagnostic accuracy to ICU clinicians; however, stratified analyses revealed clinically meaningful differences in complexity recognition and error topology.

Specifically, reduced sensitivity for mixed acid–base disorders and the presence of false reassurance classifications highlight structural limitations in physiological decomposition under high-complexity conditions.

In critical care environments, where early recognition of evolving deterioration and worst-case scenario assessment are essential to patient safety, such asymmetries in error patterns warrant careful consideration.

Because case complexity cannot be prospectively identified at the bedside and because current general-purpose large language models do not provide calibrated uncertainty signaling or escalation mechanisms, their outputs should not be considered interchangeable with physician-level interpretation in ICU settings.

The findings of this study do not establish clinical equivalence between ChatGPT and ICU physicians in acid–base interpretation. Safety-oriented evaluation frameworks — including complexity stratification, component-level analysis, and harm-weighted modeling — are necessary when assessing AI diagnostic tools in high-acuity care. Further multi-center studies with larger cohorts, longitudinal trajectory modeling, and formal calibration assessment are required before considering broader clinical integration of large language models in critical care settings.

## Supplementary Information

Below is the link to the electronic supplementary material.

Supplementary Material 1

Supplementary Material 2

## Data Availability

All relevant data are included within the article. Additional information can be obtained from the corresponding author upon request.

## Declarations

### Clinical Trial Number

A Clinical Trial Number (CTN) was not obtained as this was a non-interventional study.
