# Supplementary material for: Structural error asymmetry and harm-weighted analysis of ChatGPT versus ICU Physicians in acid–base interpretation: a prospective observational study
Source: Sci Rep. 2026 Mar 27;16:15184. doi: 10.1038/s41598-026-44576-4 (PMC13179372; doi:10.1038/s41598-026-44576-4)
Supplement: Supplementary file 1 — Supplementary Material 1 [file 41598_2026_44576_MOESM1_ESM.docx]

**Composition and Qualifications of the Blinded Expert Panel**

The blinded expert panel consisted of **three board-certified intensivists,** each actively practicing in a tertiary-level intensive care unit.

All panel members held formal specialist certification in anesthesiology and intensive care medicine and had **a minimum of 6 years of ICU clinical experience** (median experience: 12 years; range: 6–20 years).

Panel members were not involved in the direct bedside care of the included patients during case adjudication. They reviewed de-identified, standardized clinical vignettes and did not have access to patient identifiers, longitudinal trends, treatment responses, or outcomes beyond the information contained in the vignette.

Panel members independently reviewed each case and participated in a structured consensus process to determine the final reference diagnosis. Disagreements were resolved through discussion until unanimous agreement was reached.

Panel members were blinded to the identity and assessments of the initial ICU physician as well as to the ChatGPT-generated interpretations.
