# Supplementary material for: Structural error asymmetry and harm-weighted analysis of ChatGPT versus ICU Physicians in acid–base interpretation: a prospective observational study
Source: Sci Rep. 2026 Mar 27;16:15184. doi: 10.1038/s41598-026-44576-4 (PMC13179372; doi:10.1038/s41598-026-44576-4)

**Supplementary Appendix A**

All cases were derived from real ICU patients and presented as structured clinical vignettes reflecting real-world decision-making contexts rather than isolated laboratory data.

**Standardized ChatGPT Prompt and Example Clinical Input**

***A1****. Standardized ChatGPT Prompt*

The following standardized prompt was used for all ChatGPT evaluations without modification across cases:

You are an intensive care specialist. You will be given a clinical case with history, physical examination findings, laboratory values, and an arterial blood gas result. Please interpret the arterial blood gas in the context of the clinical case and provide the most likely diagnosis or clinical interpretation of the acid–base status, including identification of the primary acid–base disorder and its most likely underlying cause.

***A2****. Example of Structured Clinical Input Provided to ChatGPT (Verbatim)*

The following representative clinical vignette illustrates the standardized structure and level of detail provided to ChatGPT for each patient.

Patient ID: ICU-001

Age: 67 years

Sex: Male

Reason for ICU Admission:

Septic shock secondary to pneumonia

Respiratory Support:

Mechanical ventilation (SIMV mode; PEEP 5 cmH₂O; FiO₂ 0.40; respiratory rate 18/min; tidal volume 500 mL)

Hemodynamic Support:

Continuous norepinephrine infusion

Physical Examination:

Glasgow Coma Scale: 13 (E4V4M5)

Respiratory rate: 22/min

Blood pressure: 92/60 mmHg

Heart rate: 110 beats/min

Body temperature: 37.9°C

Lung auscultation: bilateral basal crackles

Capillary refill time: 4 seconds

Comorbidities:

Hypertension; type 2 diabetes mellitus; chronic kidney disease (stage 3)

Laboratory Results:

Hemoglobin: 10.2 g/dL

White blood cell count: 14,000/µL

Platelet count: 240,000/µL

Sodium: 135 mmol/L

Potassium: 4.5 mmol/L

Chloride: 104 mmol/L

Serum bicarbonate: 18 mmol/L

Urea: 58 mg/dL

Creatinine: 2.0 mg/dL

Glucose: 168 mg/dL

Aspartate aminotransferase (AST): 32 U/L

Alanine aminotransferase (ALT): 28 U/L

Total bilirubin: 1.2 mg/dL

Albumin: 2.8 g/dL

C-reactive protein: 120 mg/L

Lactate: 3.8 mmol/L

Arterial Blood Gas Analysis:

pH: 7.32

PaCO₂: 30 mmHg

PaO₂: 68 mmHg

Bicarbonate: 17.5 mmol/L

Base excess: −6 mmol/L

Oxygen saturation: 90%

Fraction of inspired oxygen (FiO₂): 0.40

***A3****. Methodological Note*

All clinical vignettes were generated from prospectively collected ICU data using the same standardized structure, variable set, and prompt wording. ChatGPT analyses were performed independently for each case, without access to other patient data or final clinical diagnoses.

**STROBE Statement—Checklist of Items That Should Be Included in Reports of Observational Studies**

**Study Title:**
Structural Error Asymmetry and Harm-Weighted Analysis of ChatGPT Versus ICU Physicians in Acid–Base Interpretation: A Prospective Observational Study

**TITLE AND ABSTRACT**

| **Item** | **Recommendation** | **Reported (Yes/No)** | **Location in Manuscript** |
| --- | --- | --- | --- |
| 1a | Indicate study design in title or abstract | Yes | Title; Abstract Methods |
| 1b | Provide structured, balanced summary | Yes | Abstract |

**INTRODUCTION**

| **Item** | **Recommendation** | **Reported** | **Location** |
| --- | --- | --- | --- |
| 2 | Scientific background and rationale | Yes | Introduction |
| 3 | Objectives and hypotheses clearly stated | Yes | End of Introduction |

**METHODS**

| **Item** | **Recommendation** | **Reported** | **Location** |
| --- | --- | --- | --- |
| 4 | Study design clearly described | Yes | Methods – Study Design |
| 5 | Setting, locations, dates | Yes | Methods – Study Design & Population |
| 6a | Eligibility criteria, selection method | Yes | Methods – Study Population |
| 6b | Matching criteria (if applicable) | Not applicable | — |
| 7 | Clear definition of outcomes, exposures, predictors | Yes | Methods – Variables & Diagnostic Harmonization |
| 8 | Data sources and measurement | Yes | Methods – Data Collection |
| 9 | Bias mitigation strategies | Yes | Methods – Blinding & Workflow |
| 10 | Study size explanation | Yes | Methods – Study Population |
| 11 | Handling of quantitative variables | Yes | Methods – Statistical Analysis |

**STATISTICAL METHODS**

| **Item** | **Recommendation** | **Reported** | **Location** |
| --- | --- | --- | --- |
| 12a | Statistical methods described | Yes | Methods – Statistical Analysis |
| 12b | Subgroup and interaction analyses | Yes | Methods – Complexity & Component Analysis |
| 12c | Missing data handling | Yes (no missing data) | Methods – Population Description |
| 12d | Loss to follow-up | Not applicable | — |
| 12e | Sensitivity analyses | Yes (bootstrap & harm modeling) | Methods – Harm Analysis |

**RESULTS**

| **Item** | **Recommendation** | **Reported** | **Location** |
| --- | --- | --- | --- |
| 13a | Participant flow | Yes | Results – Study Population |
| 13b | Reasons for exclusion | Yes | Methods – Exclusion Criteria |
| 13c | Flow diagram | Yes (Supplementary) | Supplementary Figure |
| 14a | Descriptive data | Yes | Results – Population Characteristics |
| 14b | Missing data | None | Results |
| 14c | Follow-up time | Not applicable | — |
| 15 | Outcome data | Yes | Results – Distribution of Diagnoses |
| 16a | Main results with effect estimates | Yes | Results – Accuracy, Kappa, Harm |
| 16b | Category boundaries clarified | Yes | Methods – Diagnostic Harmonization |
| 16c | Absolute risk measures | Yes (false reassurance rate) | Results – Mixed Detection |
| 17 | Additional analyses | Yes | Results – Component & Harm Analysis |

**DISCUSSION**

| **Item** | **Recommendation** | **Reported** | **Location** |
| --- | --- | --- | --- |
| 18 | Key results summarized | Yes | Discussion – Opening Paragraph |
| 19 | Limitations discussed | Yes | Discussion – Limitations Section |
| 20 | Interpretation cautious and contextualized | Yes | Discussion |
| 21 | Generalisability discussed | Yes | Discussion – External Validity |

**OTHER INFORMATION**

| **Item** | **Recommendation** | **Reported** | **Location** |
| --- | --- | --- | --- |
| 22 | Funding source disclosed | Yes | Declarations Section |
| — | Ethics approval | Yes | Methods – Ethics Statement |
| — | Data availability | Yes | Data Availability Section |
| — | Conflicts of interest | Yes | Declarations |

**Final STROBE Compliance Statement**

This manuscript adheres to the STROBE (Strengthening the Reporting of Observational Studies in Epidemiology) guidelines for reporting observational studies. All applicable checklist items have been addressed within the manuscript and supplementary materials.

**Supplementary Table S2** – Clinician characteristics

| **Characteristic** | **Value** |
| --- | --- |
| Number of clinicians | 3 |
| ICU background | Internal Medicine / Anesthesiology |
| Training level | Residents / Fellows / Attendings |
| Median ICU experience | 14 years (IQR) |

**Supplementary Figure S1**: study workflow

ICU admission

↓

Eligibility screening

↓

50 patients included

↓

Data extraction (ABG + clinical data)

↓

Independent interpretations

↙ ↓ ↘

ICU MD ChatGPT Expert panel

↓

Final reference diagnosis

↓

Statistical comparison

**Supplementary Figure S2**. **Distribution of initial ICU physician and ChatGPT diagnoses stratified by final ICU diagnosis.**


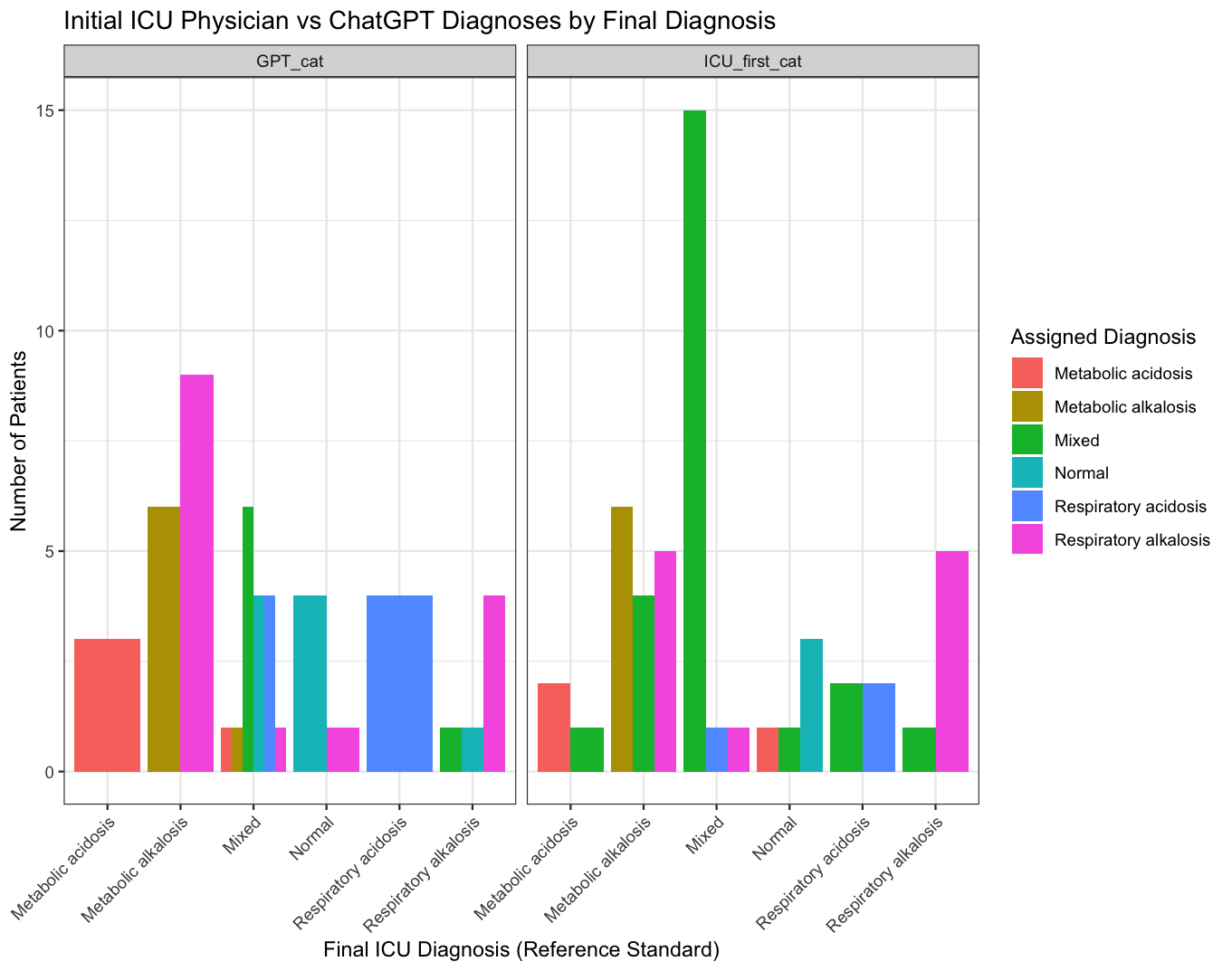

Supplement: Supplementary file 2 — Supplementary Material 2 [file 41598_2026_44576_MOESM2_ESM.docx]
